# EDITORIAL COMMENT: LAPAROSCOPIC PECTOPEXY: INITIAL EXPERIENCE OF SINGLE CENTER WITH A NEW TECHNIQUE FOR APICAL PROLAPSE SURGERY

**DOI:** 10.1590/S1677-5538.IBJU.2017.0070.1

**Published:** 2017

**Authors:** Bruno Nicolino Cezarino

**Affiliations:** 1 Serviço de Urologia do Hospital das Clínicas, Universidade de São Paulo, São Paulo, SP, Brasil

Pelvic organ prolapse (POP) affects millions of women worldwide, and is a health problem for 50% of parous women aged over 50 years (1). Multiple procedures and surgical techniques have been used, with or without the use of vaginal meshes, due to common treatment failure, reoperations, and complication rates in some studies. Randomized trials comparing the use of mesh to native tissue repair in POP surgery have now shown better anatomical but similar functional outcomes, with transvaginal meshes being associated with more complications. Surgeons so on started to use again classic techniques to correct POP using minimally invasive surgery as laparoscopy and robotic surgery (2).

Laparoscopic sacrocolpexy has been used over the time as a good option for apical prolapse correction, with some reported complications as defecation disorders due to pelvic narrowing and hypogastric nerves lesions, specially in obese populations. This retrospective analysis shows an alternative technique to correct apical prolapse, using the iliopectineal ligament (Cooper ligament) to fixate a tension free mesh to vaginal apex instead to classic sacrum fixation. In this method, the mesh follows round and broad ligaments without crossing the ureter or bowel; therefore, the pelvic outlet does not shrink. In addition, the hypogastric vessels are also a safe distance from any danger (3).

Seven patients were submitted to Laparoscopic pectopexy without intraoperative and postoperative complications. Recurrence of apical prolapse, urgency, constipation, stress urinary incontinence, anterior and lateral defect cystoceles, and rectoceles did not occur in these seven patients during the 6-month follow-up period. Despite of the inherent limitations of this retrospective study with limited number of patients, this is a promising techinique that can be used alternatively to sacrocolpopexy, specialy in obese patients or limited surgeons experience in minimally invasive approaches. Randomized controlled trials are still needed to compare results with the gold - standard procedure and to best define indications to this new technique.
